# The Significance of Atrial Fibrillation in Patients With Transient Global Amnesia

**DOI:** 10.3389/fneur.2022.830727

**Published:** 2022-03-07

**Authors:** Naaem Simaan, Asaf Honig, Andrei Filioglo, Molad Jeremy, Ronen R. Leker

**Affiliations:** ^1^Department of Neurology, Ziv Medical Center, Safed, Israel; ^2^The Azrieli Faculty of Medicine, Bar Ilan University, Safed, Israel; ^3^Department of Neurology, Hadassah-Hebrew University Medical Center, Jerusalem, Israel; ^4^Department of Neurology, Sourasky Medical Center, Tel Aviv, Israel

**Keywords:** transient global amnesia, atrial fibrillation, diffusion-weighted imaging, hippocampal lesions, micro-embolic stroke

## Abstract

**Background and Purpose:**

The etiology of transient global amnesia (TGA) remains unclear in a large subset of patients. We aimed to determine the clinical and radiological characteristics of TGA-patients with suspected acute micro-embolic stroke on diffusion-weighted imaging (DWI).

**Methods:**

TGA-patients that had new DWI hippocampal lesions (DWI+) were compared to DWI negative TGA-patients (DWI–). Demographics, risk factors, clinical data, radiological data, and mortality were analyzed.

**Results:**

Out of 83 patients diagnosed with TGA, 56 (65%) underwent MRI during the acute hospitalization and 26 (46%) had new hippocampal DWI lesions. DWI+ patients more often had a history of atrial fibrillation (AF, 26 vs. 7%, *p* = 0.04) but the frequency of other risk factors did not differ. None of the patients died, however, two DWI+ patients had subsequent stroke during a 2-year follow up and both had AF. In contrast, none of the DWI- patients had recurrent events.

**Conclusion:**

AF is common among DWI+ TGA-patients. The presence of AF in patients with TGA could suggest an increased risk of subsequent stroke.

## Introduction

Transient global amnesia (TGA) is a clinical syndrome characterized by sudden onset of anterograde and retrograde memory loss that lasts up to 24 h and is accompanied by repetitive questioning without focal neurological symptoms or signs (1).

Despite several proposed potential mechanisms, the precise pathogenesis of TGA often remains unclear (1–3). It is important to determine whether TGA could be secondary to cerebral ischemia, because uncertainty about the etiology and prognosis of TGA could lead to potentially unnecessary evaluations on the one hand and under-evaluation of stroke risk may lead to high rates of stroke recurrence on the other (3). Therefore, we aimed to determine the clinical and radiological characteristics of TGA-patients with possible acute micro-embolic stroke on diffusion-weighted imaging (DWI).

## Materials and Methods

The research was performed in accordance with the Helsinki declaration and rules of Good Clinical Practice. The research received approval of the ethics committee at Hadassah-Hebrew University Medical Center (0326–21-HMO).

Patients diagnosed with TGA according to the Hodges and Warlow criteria (2), were included in this retrospective analysis. Per institutional protocol all patients with suspected TGA were admitted to the department of Neurology and underwent telemetry for at least 48 h. Most of the patients were admitted to the stroke unit for initial evaluation and all include patients underwent evaluation by experienced stroke physicians.

For the purpose of the current study, we included patients who also had brain MRI scans during the acute admission. DWI MRI acquisitions were performed on 1.5T or 3T scanners in axial projections. To qualify as DWI restrictive lesion, a lesion had to hyper-intense on b1000 DWI and hypo-intense on the acquired diffusion coefficient (AC) maps. All MRI evaluations were performed by an experienced stroke neurologist (NS) blinded to the clinical and outcome data including ischemic recurrence. Patients with hippocampal DWI restriction (DWI+) were compared to TGA-patients without DWI lesions (DWI-). Demographics and vascular risk factors were compared. Primary clinical outcomes included functional outcomes, survival at 90 days, and the occurrence of recurrent stroke during follow up.

The MRI scans were studied for the number, size, and locations of hippocampal DWI lesions, presence of extra-hippocampal DWI, and presence and location of fluid attenuated inversion recovery (FLAIR) lesions. Subcortical FLAIR lesions were quantified according to the Fazekas score (4).

Follow up data regarding future vascular events and mortality was accrued from outpatient visits to the neurology clinic as well as from medical records.

Data analysis was performed with the statistical software SPSS version 24 (IBM USA). The two-sample *t*-test was applied for testing differences between the study groups for quantitative parameters. The chi-square test or Fisher's exact test were applied for non-liner parameters. *P*-value of 5% or less was considered statistically significant.

## Results

A total of 83 patients with TGA were admitted between 1/2016 and 1/2021 and were included in the prospective registry. Out of those, 56 (65%) had an MRI performed during the acute admission. The main reasons for not having an MRI were metallic foreign bodies or intracranial ferromagnetic clips (*n* = 18), cardiac pacemakers (*n* = 6) and claustrophobia (*n* = 2).

**Figure 1:**
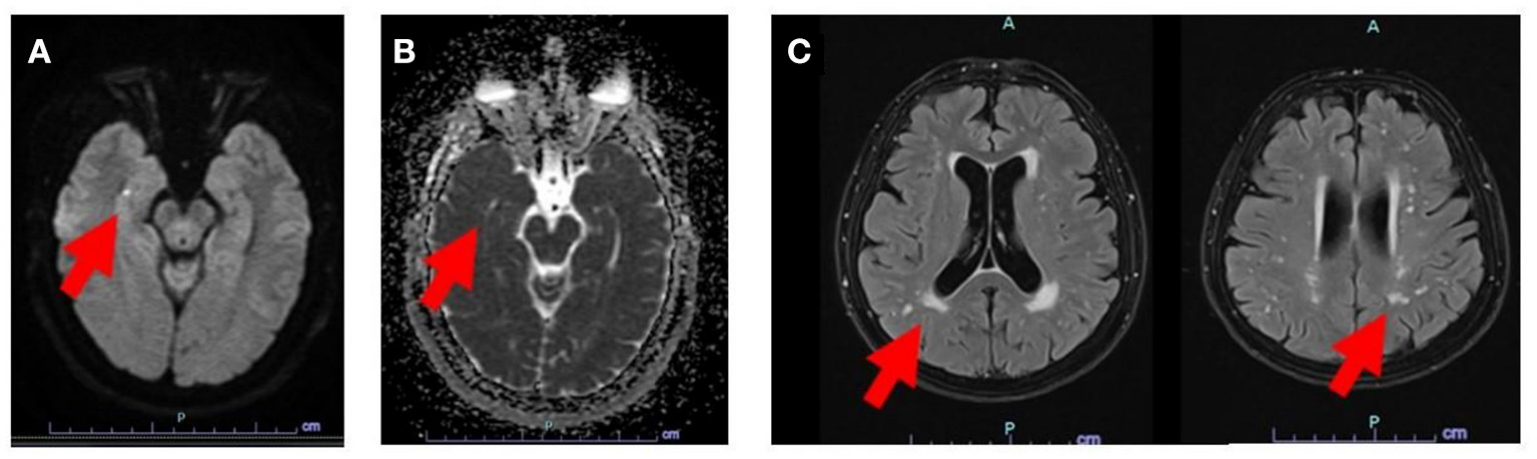
MRI image showing DWI hyperintense lesion in the right hippocampus **(A)**, also seen by the corresponding ADC maps which display diffusion restriction **(B)**. Subcortical FLAIR lesions were also found in the same patient with a Fazekas score 2 **(C)**. Red arrows point to specific lesions.

Among the 56 patients that underwent MRI (Table 1), 53 (96.5%) were screened with DWI MRI within 24–36 h from symptom onset. Out of those, 26 (46%) had new restrictive DWI lesions, all in the hippocampus (Figure 1). None of the patients had DWI lesions outside of the hippocampi. Out of the 26 DWI+ patients, 22 had unilateral lesions and 4 had bilateral hippocampal foci. The mean size (±SD) of the foci was 0.4 ± 0.1 mm. None of the patients in the DWI+ group had acute DWI restriction outside of the hippocampi. The median Fazekas score for both groups was 1.0 (IQR: 0.0–2.0, *p* = 0.818). Additionally, the mean number of FLAIR lesions was similar between the groups (Table 1). All observed FLAIR lesions were subcortical without any cortical lesions in any of the patients.

**Table 1:** Comparison of clinical parameters between patient groups with lesions seen in DWI (positive) vs. without (negative).

**Characteristics**	**• DWI positive • *N* = 27**	**• DWI negative • *N* = 30**	***P***
Age, median (IQR)	68 (63–72)	70 (62–73)	0.878
Gender male (%)	17 (63)	16 (53)	0.462
Hypertension (%)	17 (63)	18 (60)	0.819
Atrial fibrillation (%)	7 (26)	2 (7)	0.046
Diabetes (%)	6 (22)	5 (17)	0.740
Cholesterol (%)	12 (44)	15 (50)	0.675
Smoking (%)	3 (11)	3 (10)	1.0
Congestive heart failure (%)	0 (0)	3 (10)	0.239
Ischemic heart disease (%)	6 (22)	10 (35)	0.382
Chronic renal failure (%)	1 (4)	0 (0)	0.474
Prior stroke (%)	3 (11)	2 (7)	0.554
Valve (%)	1 (4)	1 (3)	1.0
Number of FLAIR lesion, mean (SD)	2.7 (2.6)	1.9 (2.3)	0.284
CHA2DS2-VASc score mean (SD)	2.83 (0.4)	1.3 (0.6)	0.003
Fazekas score, median (SD)	1.0 (0.0–2.0)	1.0 (0.0–2.0)	0.818

*Values represent number of patients unless otherwise stated. DWI, diffusion-weighted imaging*.

DWI+ patients more often had atrial fibrillation (AF) (26 vs. 7%, *p* = 0.046), the age and sex adjusted OR of AF in the DWI+: (OR 7.018, 95% CI 1.157–42.551, *p* = 0.034). Additionally, the mean CHADS_2_-VASC_2_ score was significantly higher in DWI+ group (2.83 vs. 1.3, *p* = 0.003), no other significant differences were observed between groups (Table 1).

Among the seven patients with restrictive DWI hippocampal lesions who were diagnosed with AF: two had known AF but were not on anticoagulants, three were diagnosed during admission while monitored in the stroke unit, and in one patient the AF was detected 1 month after the admission during follow up. The two patients with atrial fibrillation and no DWI lesions were diagnosed before admission and were treated with oral anticoagulants.

Follow up data was available for 56/56 of the patients (100%). None of the patients had residual disability at 3 months. Two patients (7%) from the DWI+ group had subsequent stroke during the 2-year follow up period. Both of them had AF, but only one was on oral anticoagulants at the time of the subsequent stroke. Both of them were categorized as grade 2 on the Fazekas scale, without suggestive prior embolic stroke on the FLAIR images obtained during the TGA admission. In contrast, no subsequent strokes were reported in any of the DWI^−^ patients. There were no case fatalities in either group throughout the follow-up period.

## Discussion

The main findings of the current study are that a large proportion of patients with TGA have acute DWI hippocampal lesions. We were able to demonstrate that atrial fibrillation was significantly more prevalent among patients with a new DWI lesion, who also have higher CHADS_2_-VASC_2_ score (Table 1) suggesting that TGA could be secondary to cardio-embolism in these patients. However, it should be remembered that TGA is a clinical syndrome and the diagnosis of TGA does not necessary imply that all cases are caused by the same mechanism (5–7). The fact that some patients did not have DWI lesions, and the presence of bilateral hippocampal acute lesions in four patients with AF, may point to other etiologies, since it is unlikely that these patients had isolated acute bilateral hippocampal embolic stroke. This suggests that other mechanisms including vasospasm, venous congestion, epilepsy and conversion reaction, among others, may be causative in some of the cases even among patients with AF (5–7).

In this study, the high prevalence of AF found among patients with TGA differs from previously reported studies, in which AF prevalence was similar in TGA patients and normal controls or TIA patients (8–10). However, most of these previous studies did not compare DWI+ to DWI– patients. The only other study that did compare DWI+ to DWI– patients included only 10 patients with DWI + lesions, making it prone to selection bias (11).

Additionally, in previous studies, restriction on diffusion-weighted imaging (DWI) was reported in 0% and up to 84% of TGA patients (12–15). This is probably explained by different sampling methods used in different studies (16). Furthermore, different sampling timing may have an effect on the findings. In some cases, the MRI may have been performed too late when pseudo-normalization of the DWI signal may have occurred (17). Additionally, brain remodeling may have affected the detection rate especially given the very low lesion size. In the current study, most MRI procedures were performed using a 1.5T scanner and therefore, our results may even underestimate the rate of new DWI lesions in patients with TGA. This may be due to cases of very small lesions or because of the parenchymal remolding of the lesion, which may lower the detection rate, even in follow up research with 7T MR technology (18).

The characteristics of TGA found in the current study are similar to those previously described lending further credibility to our results (9). The current study shows that the prognosis of TGA patients with a presumed vascular pathogenesis is not entirely benign with a medium risk of recurrent stroke but no fatalities observed during follow-up. Importantly, patients with recurrent stroke had AF but no evidence of previous cortical injury at the time of TGA diagnosis. Despite the low sample size limitation this may imply that TGA-patients with AF are at higher risks for future stroke and that in some patients with AF TGA may be a presenting stroke symptom. This could suggest that TGA-patients should undergo extended cardiac monitoring to rule out AF, and that DWI+ TGA episodes should count as evidence of stroke in these patients. Furthermore, the higher CHADS_2_-VASC_2_ score implying that an episode of DWI+ TGA in patients with AF should drive treatment with oral anticoagulants for stroke prevention.

The main limitations of this study are its small sample size, retrospective nature and its single-center setting. These may potentially lead to sampling errors and selection bias. Also, most MRI procedures were performed using 1.5 Tesla Scanner and therefore, our results may actually underestimate the rate of DWI lesions in TGA. However, our results offer real world data on TGA work-up and therefore has merits as hypothesis generating. Larger prospective studies are needed to corroborate our findings.

In conclusions, our data could suggest that patients presenting with TGA should be screened early with DWI MRI to rule out a potential ischemic cause. Patient with DWI hyper-intense, ADC hypo-intense lesions suggestive of ischemia should probably undergo cardiac telemetry. The prognosis of DWI+ TGA-patients is not always benign, especially in the presence of AF. Furthermore, our results suggest that TGA could be considered ischemic in nature in some DWI+ patients with AF, prompting adjustment of CHADS_2_-VASC_2_ scores accordingly and treatment with oral anticoagulants. Due to the modest sample size in the current study we believe that our results should be corroborated by larger observational studies.

## Data Availability Statement

The original contributions presented in the study are included in the article/supplementary material, further inquiries can be directed to the corresponding author.

## Author Contributions

NS and RL: study conception and design as well as data acquisition, data analysis, and drafting of the manuscript. AF: data acquirement and statistical analysis. AH and MJ: critical manuscript revision and data acquisition. All authors contributed to the article and approved the submitted version.

## Conflict of Interest

The authors declare that the research was conducted in the absence of any commercial or financial relationships that could be construed as a potential conflict of interest.

## Publisher's Note

All claims expressed in this article are solely those of the authors and do not necessarily represent those of their affiliated organizations, or those of the publisher, the editors and the reviewers. Any product that may be evaluated in this article, or claim that may be made by its manufacturer, is not guaranteed or endorsed by the publisher.
